# Decoding immune low-response states in ovarian cancer: insights from single-cell and spatial transcriptomics for precision immunotherapy

**DOI:** 10.3389/fimmu.2025.1667464

**Published:** 2025-09-19

**Authors:** Yalan Yan, Jiaan Lu, Huanyu Luo, Zizhang Wang, Ke Xu, Lexin Wang, Qin Wang

**Affiliations:** ^1^ Clinical Medical College, Southwest Medical University, Luzhou, China; ^2^ Department of Oncology, Chongqing General Hospital, Chongqing University, Chongqing, China; ^3^ Western (Chongqing) Institute for Digital-Intelligent Medicine, Chongqing, China; ^4^ Sichuan Provincial Center for Gynecology and Breast Diseases (Gynecology), Affiliated Hospital of Southwest Medical University, Luzhou, China

**Keywords:** ovarian cancer, immune low-response state, single-cell RNA sequencing, spatial transcriptomics, tumor-associated macrophages, T-cell exhaustion, immunotherapy resistance

## Abstract

Ovarian cancer represents a typically immune “cold” tumor, where obvious immunosuppression, spatial T-cell exclusion, and cellular dysfunction collectively limit immunotherapy effectiveness. Especially in high-grade serous ovarian carcinoma (HGSOC), the immune low-response state is driven by complex interactions among tumor-associated macrophages (TAMs), suppressive stromal networks, and the T-cell compartment (regulatory T cells, Tregs, and exhausted effector T cells). Emerging multi-omics technologies—particularly single-cell RNA sequencing and spatial transcriptomics—have showed the heterogeneity and spatial immune organization underlying this suppressed state. Here, we integrate these datasets to describe TAM phenotypes and spatial niches, T-cell exhaustion, Tregs accumulation, NK-cell dysfunction, and stromal barriers that enforce exclusion. We then derive phenotype-guided combination strategies to remodel the tumor microenvironment and improve responsiveness to immune checkpoint blockade. This synthesis provides a concise, multi-dimensional framework for precision immunotherapy and for overcoming resistance in immune-low ovarian cancers.

## 1 Introduction

Ovarian cancer is one of the most common gynecologic malignancies among women worldwide, with more than 320,000 cases of incidence and more than 200,000 deaths in 2022, accounting for approximately 4.0% of cancer-related deaths in women (1–4). its high lethality stems mainly from late diagnosis, heterogeneity, and poor response to standard treatment (5). Among them, high-grade serous ovarian carcinoma (HGSOC) is the most common histologic subtype with high genetic instability and complex immune escape features (6).

In recent years, although immune checkpoint blockade (ICB) has significantly improved patient prognosis in a variety of solid tumors, its therapeutic efficacy in ovarian cancer remains limited (5–8). Particularly in HGSOC, the objective remission rate of PD-1/PD-L1 monotherapy is generally low, much lower than that of immunosensitive tumors such as melanoma (8, 9). This clinical manifestation is mainly attributed to the tumor microenvironment showing a typical “immune low-response state”.

The immune low-response state is characterized by impaired antigen presentation, lack of effector T-cell infiltration, activation of immune escape pathways, and enrichment of immunosuppressive cells (e.g., TAM, Treg, and MDSC). In ovarian cancer, this state can be further subdivided into: immune-cold tumors, in which there is an almost complete lack of T-cell infiltration in the tumor tissue, reflecting the absence of antigen recognition or primitive activation signals; and immune-excluded tumors, in which T-cells are trapped in the tumor margins or stromal regions, making it difficult for them to enter the core of the tumor, which is usually associated with tumor associated fibroblasts (CAF), TGF-β signaling, and disturbed chemokine axis (10, 11).

With the rapid development of emerging multi-omics technologies such as single-cell RNA sequencing and spatial transcriptomics, researchers have been able to reveal the heterogeneity of immune cells in tumors, their genealogical trajectories, functional depletion, their spatial localization and interaction patterns with single-cell resolution, and their spatial localization and interaction patterns at single-cell resolution, providing an unprecedented opportunity to deeply analyze the nature of immune hyporesponsiveness (12–14).

The aim of this review is to systematically integrate the key studies based on scRNA-seq and spatial genomics in recent years, to comprehensively elucidate the cellular composition, spatial structure, and key signaling pathways of the immune low-response state of ovarian cancer, to identify potential therapeutic targets, and to explore multi-targeted combined immunotherapy strategies and precise subtyping methods. Through multi-omics cross-validation and immuno-mapping, we expect to provide theoretical support and translational pathways for breaking the bottleneck of drug resistance in ovarian cancer immunotherapy and promoting individualized treatment practice.

## 2 Genomic heterogeneity and immune microenvironment characteristics of ovarian cancer histologic subtypes

Ovarian cancer can be divided into several subtypes according to its histological features, including high-grade serous ovarian carcinoma (HGSOC), low-grade serous ovarian carcinoma (LGSOC), endometrioid ovarian carcinoma (EnOC), clear cell ovarian carcinoma (CCOC), mucinous ovarian carcinoma (MOC), and ovarian carcinosarcoma (OCS) (15). These histotypes exhibit marked inter- and intratumoral heterogeneity, with distinct genomic programs, immune-microenvironment features, and clinical behavior (6, 16–24).

### 2.1 The Genomic and immune landscape of HGSOC

HGSOC is the most common subtype, and almost all of them carry TP53 mutations (6, 25). p53 protein encoded by TP53 senses stress signals, such as DNA damage, and induces cell cycle arrest, senescence, apoptosis and autophagy, etc. Mutations in TP53 result in failure of the above regulatory functions, which leads to chromosomal instability (CIN) and tumor growth. Mutations lead to failure of these regulatory functions, resulting in chromosomal instability and increased tumor heterogeneity, promoting tumor progression and drug resistance (26). Approximately 50% of HGSOCs have homologous recombination repair defects (HRDs), some of which are driven by BRCA1/2 mutations (27, 28). These tumors typically have a higher neoantigenic load, a more significant tumor infiltrating lymphocyte infiltration, and are accompanied by upregulation of immune-related signals such as the PD-1-PD-L1 pathway, features that suggest they may have some immunotherapeutic potential (29–31).

However, CD8^+^ tumor-infiltrating lymphocytes (TILs) in BRCA1/2 mutant ovarian cancer were found to be more prone to enter a state of depletion, and their reactivation after anti-PD-1 treatment was relatively weak (32). In addition, it was demonstrated that PD-L1 expression is often associated with markers of immune activation (e.g., granzyme B, T-bet, and IFN-γ) as well as inhibitory markers (e.g., PD-1, CTLA-4, LAG3, etc.) were co-expressed, suggesting that immune activation and immune suppression mechanisms may co-exist in this type of tumor microenvironment, forming a complex state of immune regulation (30, 31).

On the immune map, HGSOC can also be classified as “immune cold”, “rejection” and “inflammatory”. Among them, some HGSOC are inflammatory, with abundant tumor-infiltrating lymphocytes, and may be more sensitive to immune checkpoint inhibitors (ICIs) (20, 33). However, it has been pointed out that early-stage HGSOC usually have low immunoreactivity, and present “cold” microenvironmental characteristics, suggesting that the immune status has both temporal and spatial characteristics. This suggests that the immune status is dynamically heterogeneous in time and space (34).

Recent single-cell RNA-seq in stage I HGSOC shows an immunosuppressive TME (35). FOXP3^+^ Tregs are abundant and likely suppress CD8 T cells and antigen-presenting cells through CTLA-4 with CD80/86 and through TGF-β1 signaling. Tissue-resident NK cells expressing CD103 and CD49a frequently show high NKG2A and reduced cytotoxicity. LAMP3^+^ DC and lipid-associated tumor-associated macrophages are also present. These early features support an immune-cold phenotype from tumor onset and inform the Treg- and NK-focused mechanisms and therapies (35).

### 2.2 Divergent features of non-HGSOC subtypes

In contrast, the immune profile of MOC showed a predominantly “immunocold” or “rejectionist” pattern, with sparse CD8^+^ T-cell infiltration and a low proportion of PD-L1^+^ macrophages, suggesting a lack of effective immune response potential macrophages, suggesting a lack of effective immune response potential (36). EnOC and CCOC also show a high degree of heterogeneity in molecular and immune characteristics. The mutation spectrum of the former is close to that of endometrial cancer, with common mutations such as CTNNB1, PIK3CA and ARID1A; while the latter is characterized by ARID1A deletion and high expression of HNF1B, which may be related to the immune escape and drug resistance mechanisms (37, 38).

Distinct ovarian cancer histotypes differ markedly in molecular features and also in immune reactivity and therapeutic sensitivity. This indicates that the immune-low state is not driven by a single mechanism but reflects the coevolution of tumor biology and the immune microenvironment. In recent years, single-cell transcriptomics, single-cell chromatin accessibility sequencing, and spatial transcriptomics have enabled single-cell–level dissection of the cellular composition, functional states, and spatial organization of the tumor immune microenvironment, progressively revealing the cellular and molecular underpinnings of the immune-low state in ovarian cancer (12–14). Notably, high-resolution datasets for non-HGSOC remain limited and often small, and related conclusions require validation in larger cohorts.

## 3 Immunosuppressive microenvironment and multi-omics decoding in ovarian cancer

**Figure 1:**
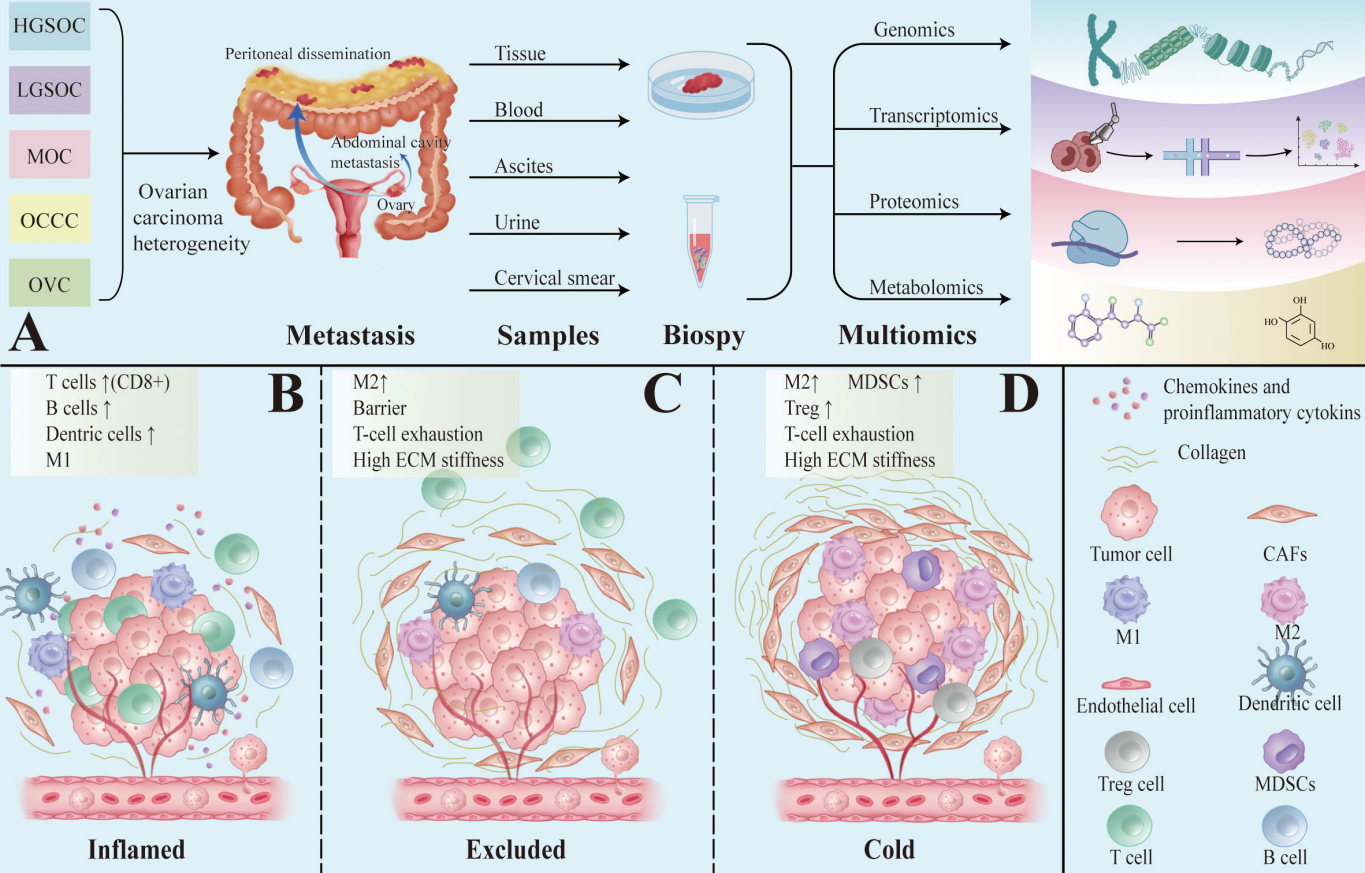
Multi-omic profiling and immune landscape of ovarian cancer. **(A)** Integration of single-cell and spatial multi-omic technologies to dissect cellular composition and spatial organization in the ovarian cancer TME. **(B)** Inflamed phenotype. **(C)** Immune-excluded phenotype **(D)** Immune-cold phenotype.

The ovarian cancer tumor microenvironment (TME) comprises malignant epithelium, stromal elements, and diverse immune lineages that vary by histotype, stage, and site. Recent single-cell and spatial multi-omics (scRNA-seq, scATAC-seq, spatial transcriptomics, proteogenomics) resolve cell identities, states, and neighborhoods at high resolution. We leverage these datasets to delineate key suppressive circuits—including TAM polarization, T-cell dysfunction with Treg accumulation, CAF-mediated barriers, and NK-cell inhibition—and to place them in spatial context (**Figure 1A**). The immune compartment spans innate cells (TAMs, dendritic cells, myeloid-derived suppressor cells, NK cells) and adaptive cells (T, B, and plasma cells).

### 3.1 Macrophage polarization

Invasion and metastasis are hallmarks of cancer. In addition to the well-recognized hematogenous and lymphatic metastatic routes, cancer cell dissemination can occur via the transcavitary route, which is typical of ovarian cancer. Macrophage TAMs are the main immunoregulatory cells of the tumor microenvironment and play a role in promoting tumor growth and dissemination to secondary sites (39–41).

The polarization status of macrophages is significantly affected by local microenvironmental factors. interferon-gamma (IFN-γ) and TNF-α induced the transformation of TAM to M1 type, which exerted pro-inflammatory and anti-tumor effects; while M2 type macrophages formed under the driving force of factors such as IL-4, IL-10, IL-13, TGF-β, and CSF1, which manifested the high expression of receptors such as CD163 and CD206. It promotes angiogenesis, stromal remodeling and tumor invasion, and recruits Tregs and suppresses effector T cells by secreting TGF-β, IL-10, CCL2, etc., constructing an immunosuppressive microenvironment (42–51). It was found that M2-type TAMs were highly enriched in HGSOC and were involved in the secretion of cytokines, such as TGF-β, IL-10, and VEGF, to promote angiogenesis, and their number was closely associated with advanced disease and poor prognosis (50–52).

M2 polarization is not only driven by cytokines, but also closely related to the metabolic status of the TME. For example, lactate released from hypoxic regions can induce differentiation of human monocytes towards M2-like phenotype and enhance their pro-tumorigenic and pro-inflammatory properties by stabilizing the autocrine circuit with CSF1 via HIF-1α signaling (53). Recent research studies also revealed that hyaluronic acid secreted by EOC cells can deplete macrophage membrane lipid raft structures via cholesterol efflux, thereby enhancing their responsiveness to IL-4, weakening the response to IFN-γ, a process that relies on STAT6 and the PI3K/AKT pathway to further drive M2 polarization (54). Izar et al. observed an increased tendency for conversion of the M1 to an M2-like state in samples from patients receiving platinum-containing chemotherapy, suggesting that treatment-associated microenvironmental changes can be malleable (55).

### 3.2 T-cell dysfunction

In addition to TAM, T cells play a key role in ovarian cancer immunosuppression (56). Although high levels of CD8^+^ tumor-infiltrating lymphocytes are often associated with a better prognosis in patients with ovarian cancer, the antitumor activity of TILs is severely suppressed in most patients.

Studies have shown that T cells in TME generally exhibit a depleted phenotype, as evidenced by persistently high expression of co-inhibitory receptors such as PD-1, LAG-3, and TIM-3, and functional loss of cytotoxicity (e.g., IFN-γ, TNF-α, and IL-2) along with a loss of proliferative capacity (57). The molecular features of these TILs involve impairments in TCR signaling (e.g., PD-1 blockade of Lck-mediated ZAP70 phosphorylation) and activation of depletion-associated programs driven by multiple transcription factors (e.g. TOX, NR4A, IRF2, etc.) (58–63). DNA methylation modifications also accelerate the formation of the terminal depletion state, and DNMT3A-mediated epigenetic reprogramming limited their responsiveness to ICIs (64, 65).

In addition, a portion of TILs entered an irreversible senescence state, accompanied by phenotypes such as telomere shortening and enhanced Senescence-associated β-gal expression, which further weakened their response capacity. The formation mechanism may be related to the continuous stimulation by tumor antigens and pro-inflammatory factors (e.g., IL-6, TNF-α) (66–68).

Furthermore, T cell recruitment from the periphery to tumor tissue is limited by multiple barriers, particularly in “immune-excluded” tumors, where successful T cell homing and infiltration of the tumor parenchyma is dependent on the presence of a “permeable” vascular endothelium. The successful homing of T cells and infiltration of tumor parenchyma is dependent on the presence of “permeable” vascular endothelium. However, in immune-excluded TME, these series of cell adhesion molecules (e.g., selectin, ICAM-1, VCAM-1, etc.) are often down-regulated or spatially impaired, significantly impairing T-cell adhesion and exocytosis (69, 70).

Tregs and MDSCs, as major immunosuppressive cell populations, also play a key role in the maintenance of T cell dysfunction. Tregs inhibit effector T cell activation through mechanisms such as the expression of CTLA-4 and the secretion of TGF-β and IL-10, while MDSCs interfere with T cell proliferation and effector function through multiple pathways such as the depletion of L-arginine, secretion of reactive oxygen/nitrogen species, etc (71). A study has identified a novel mechanism by which MDSCs further induce CD8^+^ T-cell functional decline through the GPR84 signaling axis. This study found that GPR84 can be transferred from MDSCs to CD8^+^ T cells via exosomes (Exosomes), which in turn activate the p53 signaling pathway and induce them to enter a senescent state. Impaired proliferative capacity and functional decline were observed in GPR84 overexpressing CD8^+^ T cells, whereas knockdown of GPR84 or p53 partially restored T cell effector function (71). Transcriptome analyses further confirmed that treatment of GPR84^+^ MDSCs significantly enhanced the proliferative capacity of CD8 ^+^T cell activity of the p53-related pathway in T cells, which is closely related to their phenotypic senescence (71).

Tregs accumulate in HGSOC primary tumors and malignant ascites and suppress antitumor immunity via the CTLA-4–CD80/CD86 axis and IL-10/TGF-β–mediated pathways. The follicular regulatory subset (Tfr; CXCR5^+^CD25^+^FOXP3^+^) is increased in ovarian cancer; in CD8–Tfr co-cultures, Tfr cells inhibit CD8^+^ T-cell activation in an IL-10–dependent manner with supportive evidence for TGF-β cooperation (72). In malignant ascites from epithelial ovarian cancer, effector-type Tregs are increased, positively associate with CD8^+^ PD-1, and frequently express CCR4—supporting a Treg-enriched, checkpoint-high suppressive milieu and pointing to the CCL22/CCL17–CCR4 axis as a recruitable, and potentially targetable, node (73).These observations support the immune-low phenotype of HGSOC and help explain the modest activity of PD-1/PD-L1 monotherapy.

T-cell dysfunction stems not only from exogenous intervention of suppressor cell populations, but also from an imbalance in T-cell self-regulation in the context of continuous antigenic stimulation and a harsh metabolic environment. As a result, ICIs therapies targeting co-inhibitory molecules are often difficult to reverse the deep depletion state, suggesting that more precise therapeutic strategies, such as metabolic reprogramming, epigenetic regulation, and multi-targeted interventions, need to be explored in order to improve the clinical benefits of immunotherapy for ovarian cancer.

### 3.3 CAF

Cancer-associated fibroblasts, as a key immunoregulatory hub in TME, are widely involved in extracellular matrix (ECM) remodeling, tumor cell migration, angiogenesis, and immune regulation (74). In recent years, single-cell RNA sequencing has revealed that CAFs are highly heterogeneous, and can be subdivided into myofibroblastic CAFs, inflammatory CAFs, iCAFs, and other functions. iCAFs) and other functional subgroups, among which there are significant differences in molecular markers, spatial distribution and immune functions.

Izar et al. identified four subpopulations of CAFs based on scRNA-seq analysis of ascites-derived HGSOC samples, two of which were enriched in immune-related factors such as CXCL1/2/10/12, IL6 and IL10, suggesting that they have immunomodulatory functions. These CAFs are referred to as “inflammatory CAFs” or “iCAFs”, which promote tumor immune escape and drug resistance through the secretion of cytokines, recruitment of immune cells, and activation of the JAK/STAT pathway. For example, IL6 secreted by CAFs activates the STAT3 signaling pathway in cancer cells and macrophages, enhancing pro-inflammatory properties and suppressing anti-tumor immune responses (55).

The JAK/STAT signaling axis is a key pathway in the ligand-receptor interactions between CAF, cancer cells and immune cells. The secretion of IL6 and CXCL12 by CAF activates the JAK1/2-STAT3/5 pathway in cancer cells and enhances their proliferative and invasive abilities, while cancer cells themselves express a variety of downstream inflammation-related genes (e.g., IL6, TNF, IFI6, ISG15, etc.), forming self-excitation or positive feedback loops (75). The activation of this pathway is thought to be an important mechanism of tumor heterogeneity and immune tolerance, and in the PDX model established by Izar et al. the JAK/STAT inhibitor JSI-124 significantly inhibited tumor growth and ascites formation, suggesting it as a potential therapeutic target (55).

CAFs shape the “immune cold” state through inflammatory factor secretion, cellular interactions, and signaling axis activation, which not only promotes tumor progression but also significantly affects the response to immunotherapy. In the future, functional typing and targeted intervention strategies based on the characteristics of CAF subgroups may provide new ideas for improving immunotherapy response in ovarian cancer patients.

### 3.4 NK-cell

In HGSOC ascites and primary sites, CD103^+^/CD49a^+^ tissue-resident NK cells (trNK) and CD8^+^ T cells are present; trNK frequently exhibit high NKG2A and display robust ex vivo cytotoxicity against ovarian tumor targets (76). This patient-derived evidence indicates that innate effectors are present but restrained by checkpoints and the microenvironment, which—together with T-cell exhaustion and stromal barriers—sustain the immune-low state. It also motivates NKG2A-directed checkpoint release in combination with stromal modulation.

## 4 Spatial structure and signaling mechanism of the immune low-response state of ovarian cancer

The immune cold or immune excluded phenotypes of ovarian cancer are not only reflected in the changes of immune cells, but also rooted in the remodeling of the spatial structure of the tumor microenvironment (TME) and the cellular communication mechanism at a deeper level. In recent years, with the help of multi-omics technology and spatial transcriptome studies, researchers have revealed key mechanisms such as restricted immune cell recruitment, suppression of effector functions, and aberrant activation of signaling axes, which provide clues for an in-depth understanding of the nature of immune hyporesponsive states.

### 4.1 Spatial heterogeneity and immunosuppressive cell localization

Ovarian cancer TME shows obvious spatial heterogeneity, which can be broadly categorized into three types: “inflamed” (**Figure 1B**), “excluded” (**Figure 1C**) and “cold” (**Figure 1D**).

In immune-excluded ovarian cancer, CD8^+^ tumor-infiltrating lymphocytes (TILs) are primarily retained in the stromal region and have difficulty penetrating the epithelial region of the tumor. The researchers suggest that this restricted spatial distribution may be caused by a combination of impaired permeability of the tumor vascular endothelium, dense extracellular matrix (ECM) deposited by CAFs, and mechanical barriers such as those constructed by TREM2^+^M2-type macrophages (70, 77).

Impaired endothelial cell activation is a typical manifestation of rejection-type TME. In the normal immune response, proinflammatory cytokines induce endothelial cells to express adhesion molecules such as selectins, ICAM-1, and VCAM-1, which form the activation plaques required for “T-cell homing” (78–81). PD-1/PD-L1 and CD80/PD-L1 signaling axis not only act as immune checkpoints, but also directly regulate the migration of Treg and Teff across the lymphatic or vascular endothelium in tumors. This signaling pathway mediates VE-cadherin junction stability and endothelial VCAM-1 expression through PI3K/Akt, ERK, and NF-κB-p65, thereby affecting T cell adhesion and crossing behavior (82). Blockade of PD-1 or CD80 not only impairs T cell migration, but also promotes tumor immune infiltration and tumor control in *in vivo* models. In addition, prostaglandin E2 (PGE2), vascular endothelial growth factor A (VEGF-A), and endothelin-β secreted by tumor cells synergistically induced vascular endothelial cells to overexpress FAS ligand (FASL), which selectively induced effector T cells (e.g., CD8^+^T cells (e.g., CD8^+^T cells) through the FAS-FASL pathway, whereas Tregs were virtually unaffected (69, 70, 83, 84). This mechanism of selective exclusion not only prevented tumor-specific effector T cells from penetrating the vascular barrier to enter the tumor parenchyma but also maintained immunosuppressive mechanisms such as Tregs. This “selective rejection” mechanism not only prevented tumor-specific effector T cells from penetrating the vascular barrier into the tumor parenchyma, but also maintained the enrichment of immunosuppressive cells, such as Tregs, which further solidified the immunosuppressive state of rejected TMEs (69, 85).

CAF constructs a matrix structure with high rigidity and low permeability by secreting factors such as TGF-β, VEGF, and CXCL12, and enhancing the expression of collagen fiber remodeling genes (e.g., COL11A1, COMP, and FN1) (55, 86). This type of “de-activated” matrix not only hinders the migration and penetration of effector T cells, but may also limit their chemotactic recruitment through the formation of chemical gradients.

### 4.2 Immune signaling axis and cellular communication mechanisms

**Figure 2:**
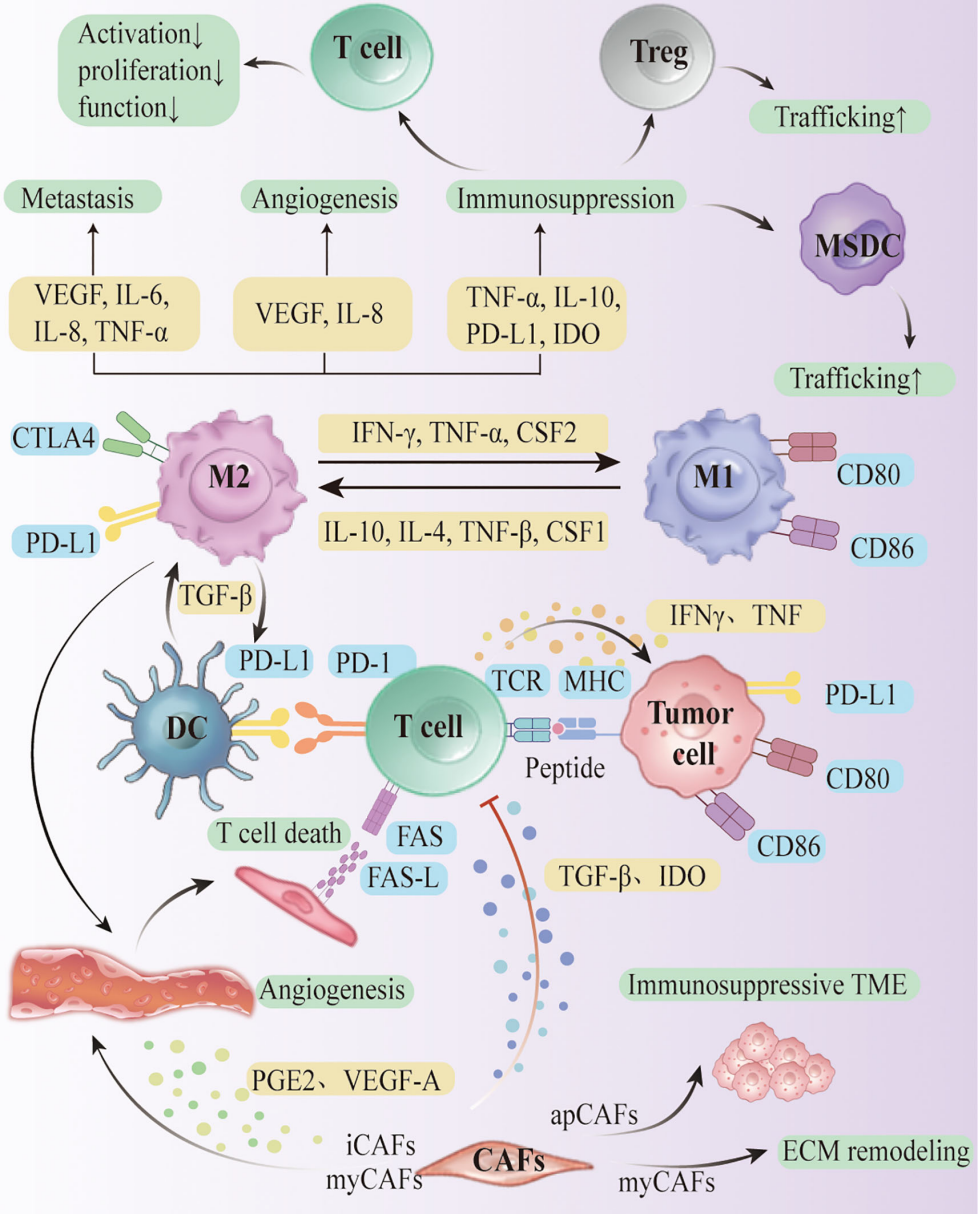
Cellular interactions within the ovarian cancer TME.

The immune signaling axis in the tumor microenvironment (TME) of ovarian cancer builds a complex intercellular communication network, which plays a key role in the formation of the immune low-responsive state (**Figure 2**). Several studies have shown that typical immune signaling pathways, such as CXCL12-CXCR4, VEGF-VEGFR, IL-6-JAK-STAT3, and TGF-β-TGFβR, are They are widely present in the interactions among cancer cells, CAFs, TAMs, MDSCs, Tregs and TILs, and regulate the homing, differentiation, depletion and rejection of immune cells, thus solidifying the “rejection-type” and “desert-type” immune phenotypes. The “rejection” and “desert” immune phenotypes (87). Coagulation-linked gene signatures in melanoma track immune infiltration and prognosis, suggesting that coagulation–inflammation coupling may also modulate the ovarian cancer TIME and merits targeted evaluation (88).

In particular, cancer-associated fibroblasts (CAFs) secrete a variety of chemokines and cytokines that synergistically induce the expression of FASL in vascular endothelial cells, mediate selective apoptosis of effector CD8^+^ T cells, and promote the enrichment of Tregs, MDSCs, and other cells (89). In addition, the PGE2-EP2/EP4 axis can play an important role in chemotherapy and other therapeutic treatments. EP4 axis can be upregulated after genotoxic stresses such as chemotherapy, inducing iron death (ferroptosis) in depleted TILs and inhibiting their effector differentiation capacity, exacerbating immune clearance deficits (85, 90).

Notably, recent studies have revealed that ovarian cancer stem cells (OCSCs) not only possess strong immune escape ability, but also actively participate in the construction of the immune signaling axis by interacting with TAMs, CAFs and the hypoxic microenvironment.Cancer stem cells induced TAMs to secrete factors such as IL-6, IL-8, and TGF-β, which activated the STAT3 and SMAD pathways, maintained their stemness and promoted the recruitment of immunosuppressive cells (91–93). Meanwhile, WNT5a and IL-6 secreted by CAFs activated the ROR2/PKC and JAK/STAT3 axis, enhancing the self-renewal and immune escape ability of OCSCs (93, 94). In addition, OCSCs themselves highly express PD-L1 and immunosuppressive factors (e.g., IDO1, CCL5, CXCL2), which can recruit Tregs, inhibit the function of TIL, and construct an “immune cold” microenvironment (93). “Combining the latest findings from single-cell and spatial transcriptomic data, OCSCs are considered to be the coordinators of multiple signaling axes in immune hyporesponsive states, and their spatial localization and functional activity are gradually becoming potential targets for precision immunotherapy (95).

Interactions between NK cells and myeloid populations can constrain NK effector function and facilitate immune escape. In ascites and primary sites, CD103^+^/CD49a^+^ tissue-resident NK cells frequently express the inhibitory receptor NKG2A, consistent with a “present-but-restrained” NK state (76). Dendritic cells can further modulate NK activation via checkpoint/ligand engagement and soluble mediators; in particular, DC-derived PGE_2_ and IL-10 act as negative regulators that limit NK activation and migration (96).

Beyond cytokines and chemokines, extracellular vesicles also mediate cell–cell communication: in hepatocellular carcinoma, exosomes reprogram TAMs, DCs and T cells and reshape the immune microenvironment; analogous vesicle-mediated pathways likely operate in ovarian cancer and warrant direct evaluation (97).

The immune signaling axis in ovarian cancer is not only the basis of cellular communication, but also works together to maintain a complex immune escape program through network interactions with components such as cancer stem cells, immunosuppressive cells, and CAF. The high heterogeneity and dynamic plasticity of this network structure determines the limitations of single-target intervention strategies, suggesting that multi-target blockade, synergistic signaling axes and other precise strategies are needed to break the immune hyporesponsive state and achieve a more effective therapeutic response.

## 5 Exploration of tumor microenvironment as a combination therapy

The treatment of ovarian cancer mainly includes surgery combined with platinum-containing chemotherapy, and some patients with BRCA mutation or HRD positivity can receive PARP inhibitors (e.g., Olaparib) for maintenance therapy (20, 98–102). The antiangiogenic drug Bevacizumab is also used in patients with recurrent or advanced disease (103). Immune checkpoint inhibitors have shown good efficacy in a wide range of tumors, but are ineffective as single agents in ovarian cancer, with an objective remission rate of less than 15%. Studies have shown that ovarian cancer is often characterized by “immune hyporesponsiveness”, such as low TIL infiltration and enrichment of immunosuppressive cells, which significantly limits the application of ICIs.

Therefore, current research focuses on combination therapy strategies to remodel TME and reverse the immunosuppressive state to enhance immunotherapy response. In particular, TME characterization based on single-cell and spatial profiling is driving the exploration of individualized combinations of multiple target classes and pathways (**Table 1**).

**Table 1:** Combination immunotherapy strategies for immune low-response ovarian cancer.

Mechanism type		Representative targets	Interventions	Combination therapy strategies and indications	Stage	Status	Identifier	Reference
Tumor-Associated Macrophages (TAMs)	Blocking TAM recruitment	CSF1-CSF1R	Cabiralizumab	Cabiralizumab in combination with Navulizumab in advanced solid tumors.	Phase I	Completed	NCT02526017	
			Emactuzumab (RO 5509554) (RG-7155)	Paclitaxel and bevacizumab ± emactuzumab for platinum-resistant ovarian/tubal/primary peritoneal cancers	Phase II	Terminated	NCT02923739	
				RO5509554 Paclitaxel alone or in combination for advanced solid tumors	Phase I	Completed	NCT01494688	(104)
			Pexidartinib (PLX3397)	PLX3397 in combination with pembrolizumab for advanced melanoma and other solid tumors	Phase I/II	Terminated	NCT02452424	
	TAM Clearance	TRAILR	Trabectedin	Trabectedin in combination with bevacizumab ± carboplatin in advanced ovarian cancer.	Phase II	Completed	NCT01735071	(105)
	Re-education/polarized TAM	TLR	852A	TLR Agonist for the Treatment of Breast, Ovarian, Endometrial, and Cervical Cancers	Phase II	Completed	NCT00319748	
			Motolimod (VTX-2337)	VTX-2337 in combination with PLD for recurrent/persistent epithelial ovarian/tubal/primary peritoneal cancer	Phase II	Completed	NCT01666444	(106)
		PI3Kγ	IPI-549	AB928 in combination with PLD ± IPI-549 for advanced TNBC or ovarian cancer	Phase I	Completed	NCT03719326	
		CD40	CDX-1140	CDX-1140 alone or in combination with CDX-301, pembrolizumab, or chemotherapy for advanced malignancies	Phase I	Completed	NCT03329950	
	Promotes TAM phagocytosis	CD47-SI	Magrolimab (Hu5F9-G4).	Magrolimab in Combination with Avilimumab for Progressive Ovarian Cancer and Other Solid Tumors (Progression within 6 Months Post-Platinum)	Phase I	Completed	NCT03558139	(107)
		CD40	SL-172154 (SIRPα-Fc-CD40L)	SL-172154 for Ovarian Cancer	Phase I	Completed	NCT04406623	
	Inhibits the tumor-promoting function of TAM	VEGF-VEGFR	Bevacizumab	TSR-042 Combining Bevacizumab with Niraparib in Recurrent Ovarian Cancer	Phase II	Completed	NCT05751629	
				Preoperative Chemotherapy ± Bevacizumab for Advanced Ovarian Cancer	Phase II	Completed	NCT01847677	
		IDO1	INCB024360 (epacadostat)	CDX-1401 in combination with Poly-ICLC and INCB024360 in patients with NY-ESO-1/LAGE-1 positive epithelial ovarian/tubal/peritoneal cancer in remission.	Phase I/II	Completed	NCT02166905	
T-cell dysfunction	Delay/reverse T-cell depletion	PD-1	Nivolumab	WT1 or NY-ESO-1 vaccine in combination with Nivolumab for recurrent ovarian cancer	Phase I	Completed	NCT02737787	
				ACT in combination with checkpoint inhibitors for metastatic ovarian cancer	Phase I/II	Completed	NCT03287674	
			Pembrolizumab (MK-3475)	Pembrolizumab Monotherapy in Subjects With Advanced Recurrent Ovarian Cancer	Phase II	Completed	NCT02674061	(108)
				Pembrolizumab Combined With PLD For Recurrent Platinum Resistant Ovarian, Fallopian Tube Or Peritoneal Cancer	Phase II	Completed	NCT02865811	(109)
			Atezolizumab	Atezolizumab With Neoadjuvant Chemotherapy for Patients With Newly-Diagnosed Advanced-Stage Ovarian Cancer	Phase I/II	Completed	NCT03394885	
				Atezolizumab Versus Placebo in Combination With Paclitaxel, Carboplatin, and Bevacizumab in Participants With Newly-Diagnosed Stage III or Stage IV Ovarian, Fallopian Tube, or Primary Peritoneal Cancer	Phase III	Completed	NCT03038100	(110, 111)
		CTLA-4	Ipilimumab	Combination of Nivolumab and Ipilimumab in Breast, Ovarian and Gastric Cancer Patients	Phase II	Terminated	NCT03342417	
				TIL Therapy in Combination With Checkpoint Inhibitors for Metastatic Ovarian Cancer	Phase I/II	Completed	NCT03287674	
	Inhibits T-cell senescence	p38 MAPK	LY2228820	LY2228820 Combination of gemcitabine and carboplatin in platinum-sensitive ovarian cancer	Phase I/II	Completed	NCT01663857	(112)
	T-cell metabolic reprogramming	AMPK activation	Metformin	Metformin in combination with carboplatin/paclitaxel in advanced ovarian cancer (OVMET)	Phase I	Completed	NCT02312661	

### 5.1 Tumor-associated macrophages as combination therapy targets

TAMs are one of the most abundant immunosuppressive cells in ovarian cancer TME, which mainly exhibit M2-like phenotype and are involved in immune escape, angiogenesis, tumor cell migration and drug tolerance. In recent years, multiple therapeutic strategies targeting TAM have been proposed, including recruitment blockade, depletion and clearance, phenotypic re-education, promotion of phagocytosis restoration and signaling pathway modulation, and some of these strategies have entered clinical trials.

TAM recruitment is dependent on the CSF1/CSF1R signaling axis, and several monoclonal or small molecule inhibitors (e.g., pexidartinib) have demonstrated good TAM removal and immune activation effects in trials (113, 114). IPI-549, a selective inhibitor of PI3Kγ, also blocks TAM recruitment and polarization, restores CTL activity and produces synergistic effects in combination with ICIs.211 The PI3Kγ-selective inhibitor IPI-549 also blocks TAM recruitment and polarization, restores CTL activity and produces synergistic effects with ICIs. ICIs to produce synergistic effects in combination (115). TLR agonists (e.g., TLR7 agonist 852A, TLR8 agonist motolimod), although with limited efficacy as monotherapies in EOC, induce local immune activation and enhance the potential for ICIs response (116, 117).

TAM acts as an antigen-presenting cell and its phagocytosis is inhibited by CD47 and CD24. Blocking the CD47-SIRPα axis significantly enhances macrophage-mediated tumor phagocytosis, which has been shown to be effective in EOC models, and several anti-CD47 monoclonal antibodies (e.g., magrolimab) are in clinical trials (107). However, due to the widespread expression of CD47, a new generation of bispecific antibodies and fusion proteins are being developed to reduce side effects and improve specificity.

In addition to clearance and re-education, immunosuppressive factors secreted by TAM can also be directly targeted. IDO is a key metabolic enzyme, and inhibition of its expression restores CD8^+^T and NK cell function. Although IDO inhibitors (e.g., epacadostat) failed phase II trials in EOC, combining them with A2aR antagonists demonstrated metabolic reversal potential, providing new ideas for future combination therapy.

TAM expresses immune checkpoint molecules such as PD-L1, which can limit T cell activation. Although anti-PD-L1 monoclonal antibodies have limited efficacy in EOC alone, combining them with TAM-targeted drugs, PARP inhibitors, and anti-angiogenic drugs significantly enhances therapeutic response. For example, durvalumab in combination with olaparib has boosted TIL infiltration and remission rates in multiple EOC trials and is now entering the phase III trial DUO-O (NCT03737643) to validate its maintenance therapy effect.

### 5.2 Immunotherapeutic strategies targeting T-cell depletion and senescence

T-cell dysfunction is one of the core mechanisms of limited response to immunotherapy, which mainly consists of T-cell depletion and senescence, which are commonly characterized by decreased cellular function, up-regulation of inhibitory receptor expression (e.g., PD-1, TIM-3), and metabolic imbalance (11).

To slow the depletion process, one strategy is to reduce the antigenic load, e.g., by attenuating sustained antigenic stimulation with chemotherapy, targeted therapy, or radiotherapy, which helps to preserve T-cell activity and enhance ICIs response.

Targeting functional recovery of depleted T cells, metabolic reprogramming is considered a key breakthrough. It has been found that TILs in ovarian cancer are often accompanied by mitochondrial dysfunction and aberrant upregulation of oxidative phosphorylation (OXPHOS), which in turn induces SIRT1 activation and accumulation of metabolic stresses.OXPHOS inhibitors and SIRT1 inhibitors can reverse TIL depletion by regulating cellular metabolic pathways. Beyond mitochondrial OXPHOS and SIRT1, stress and regulated cell-death programs also track immune contexture and therapy response in other cancers. Necroptosis-related gene sets in melanoma, disulfidoptosis-related lncRNA models in colon cancer, and an aggrephagy-related lncRNA signature in pancreatic cancer all stratify prognosis and immune features. These cross-cancer signals suggest that proteostasis and RCD pathways may shape T-cell dysfunction and immunotherapy benefit. Ovarian cancer–specific modeling and prospective validation are warranted before clinical translation (118–120).

In addition, engineered T-cell therapies are slowing down the aging and depletion process by modifying signaling pathways and epigenetic states. In the future, TCR-T and CAR-T products incorporating anti-depletion molecular modules are expected to enhance the durable immune effects in ovarian cancer treatment (121). In immune-low HGSOC, releasing NKG2A-mediated inhibition on tissue-resident NK cells together with stromal remodeling may augment responses to PD-1/PD-L1 therapy (76). Notably, single-cell data from stage-I HGSOC indicate that a Treg-rich suppressive milieu with dysfunction of CD8 T cells, NK cells, and DCs is already established, suggesting that microenvironment-remodeling strategies may need to be considered earlier in the disease course (35).

## 6 Conclusion

The state of immune hyporesponsiveness in ovarian cancer significantly limits its therapeutic efficacy. This article reviews the immune escape mechanisms of immune cells such as TAMs and depleted T cells, and summarizes the current major targeted and combination therapy strategies. With the development of single-cell histology and spatial transcriptome technologies, we have gained a deeper understanding of the complex composition and immunoregulatory network of TME, and have provided a new direction for precision immune intervention (122).

Future studies should further integrate single-cell histology, spatial transcriptome and TCR/BCR profile tracking to clarify the functional status and dynamic evolutionary pathways of different subtypes of immune cells; meanwhile, strengthen the design of multi-targeted combinatorial therapies to break through the current bottleneck of the efficacy of ICIs in ovarian cancer. Precise typing of tumor microenvironment and individualized immune intervention will be the key breakthrough to enhance the long-term survival of ovarian cancer (95). Given that immune composition can vary by disease stage and sampling site, conclusions should be interpreted in that context. Prospective studies and clinical trials ought to stratify by stage and sampling site and tailor interventions accordingly, with the goal of overcoming resistance and achieving durable survival benefits.

## References

[1] Bray F Laversanne M Sung H Ferlay J Siegel RL Soerjomataram I. Global cancer statistics 2022: GLOBOCAN estimates of incidence and mortality worldwide for 36 cancers in 185 countries. CA Cancer J Clin. (2024) 74:229–63. doi: 10.3322/caac.21834, PMID: 38572751

[2] Armstrong DK Alvarez RD Backes FJ Bakkum-Gamez JN Barroilhet L Behbakht K. NCCN guidelines^®^ Insights: ovarian cancer, version 3.2022. J. Natl Compr Canc Netw. (2022) 20:972–80. doi: 10.6004/jnccn.2022.0047, PMID: 36075393

[3] Liu J Berchuck A Backes FJ Cohen J Grisham R Leath CA. NCCN guidelines^®^ Insights: ovarian cancer/fallopian tube cancer/primary peritoneal cancer, version 3.2024. J Natl Compr Canc Netw. (2024) 22:512–9. doi: 10.6004/jnccn.2024.0052, PMID: 39413835

[4] Webb PM Jordan SJ. Global epidemiology of epithelial ovarian cancer. Nat Rev Clin Oncol. (2024) 21:389–400. doi: 10.1038/s41571-024-00881-3, PMID: 38548868

[5] Armstrong DK Alvarez RD Bakkum-Gamez JN Barroilhet L Behbakht K Berchuck A. Version 2.2020, NCCN clinical practice guidelines in oncology. J Natl Compr Canc Netw. (2021) 19:191–226. doi: 10.6004/jnccn.2021.0007, PMID: 33545690

[6] Kordowitzki P Lange B Elias KM Haigis MC Mechsner S Braicu IE. Transforming treatment paradigms: Focus on personalized medicine for high-grade serous ovarian cancer. CA Cancer J Clin. (2025) 16(10):e70789. doi: 10.3322/caac.70008, PMID: 40252048 PMC12432820

[7] Bareche Y Kelly D Abbas-Aghababazadeh F Nakano M Esfahani PN Tkachuk D. Leveraging big data of immune checkpoint blockade response identifies novel potential targets. Ann Oncol. (2022) 33:1304–17. doi: 10.1016/j.annonc.2022.08.084, PMID: 36055464

[8] Wan C Keany MP Dong H Al-Alem LF Pandya UM Lazo S. Enhanced efficacy of simultaneous PD-1 and PD-L1 immune checkpoint blockade in high-grade serous ovarian cancer. Cancer Res. (2021) 81:158–73. doi: 10.1158/0008-5472.CAN-20-1674, PMID: 33158814 PMC7878408

[9] Zhong Y Wang Y Wang C Cao K Wang X Xu X. Targeting mesothelin-CD24 axis repolarizes tumor-associated macrophages to potentiate PD-1 blockade therapy in high-grade serous ovarian cancer. J Immunother Cancer 13. (2025) 13(2):e011230. doi: 10.1136/jitc-2024-011230, PMID: 40010770 PMC11873354

[10] Herrera FG Ronet C Ochoa de Olza M Barras D Crespo I Andreatta M. Low-dose radiotherapy reverses tumor immune desertification and resistance to immunotherapy. Cancer Discov. (2022) 12:108–33. doi: 10.1158/2159-8290.CD-21-0003, PMID: 34479871 PMC9401506

[11] Ghisoni E Morotti M Sarivalasis A Grimm AJ Kandalaft L Laniti DD. Immunotherapy for ovarian cancer: towards a tailored immunophenotype-based approach. Nat Rev Clin Oncol. (2024) 21:801–17. doi: 10.1038/s41571-024-00937-4, PMID: 39232212

[12] Qian J Olbrecht S Boeckx B Vos H Laoui D Etlioglu E. A pan-cancer blueprint of the heterogeneous tumor microenvironment revealed by single-cell profiling. Cell Res. (2020) 30:745–62. doi: 10.1038/s41422-020-0355-0, PMID: 32561858 PMC7608385

[13] Zheng X Wang X Cheng X Liu Z Yin Y Li X. Single-cell analyses implicate ascites in remodeling the ecosystems of primary and metastatic tumors in ovarian cancer. Nat Cancer. (2023) 4:1138–56. doi: 10.1038/s43018-023-00599-8, PMID: 37488416 PMC10447252

[14] Wu X Yang X Dai Y Zhao Z Zhu J Guo H. Single-cell sequencing to multi-omics: technologies and applications. biomark Res. (2024) 12:110. doi: 10.1186/s40364-024-00643-4, PMID: 39334490 PMC11438019

[15] McCluggage WG Singh N Gilks CB. Key changes to the World Health Organization (WHO) classification of female genital tumours introduced in the 5th edition (2020). Histopathology. (2022) 80:762–78. doi: 10.1111/his.14609, PMID: 34996131

[16] Duska LR Kohn EC. The new classifications of ovarian, fallopian tube, and primary peritoneal cancer and their clinical implications. Ann Oncol. (2017) 28:viii8–viii12. doi: 10.1093/annonc/mdx445, PMID: 29232468 PMC6246280

[17] Barnes BM Nelson L Tighe A Burghel GJ Lin IH Desai S. Distinct transcriptional programs stratify ovarian cancer cell lines into the five major histological subtypes. Genome Med. (2021) 13:140. doi: 10.1186/s13073-021-00952-5, PMID: 34470661 PMC8408985

[18] Peres LC Cushing-Haugen KL Köbel M Harris HR Berchuck A Rossing MA. Invasive epithelial ovarian cancer survival by histotype and disease stage. J Natl Cancer Inst. (2019) 111:60–8. doi: 10.1093/jnci/djy071, PMID: 29718305 PMC6335112

[19] Siegel RL Giaquinto AN Jemal A. Cancer statistics, 2024. CA Cancer J Clin. (2024) 74:12–49. doi: 10.3322/caac.21820, PMID: 38230766

[20] Kandalaft LE Dangaj Laniti D Coukos G. Immunobiology of high-grade serous ovarian cancer: lessons for clinical translation. Nat Rev Cancer. (2022) 22:640–56. doi: 10.1038/s41568-022-00503-z, PMID: 36109621

[21] Zwimpfer TA Tal O Geissler F Coelho R Rimmer N Jacob F. Low grade serous ovarian cancer - A rare disease with increasing therapeutic options. Cancer Treat Rev. (2023) 112:102497. doi: 10.1016/j.ctrv.2022.102497, PMID: 36525716

[22] Driva TS Schatz C Haybaeck J. Endometriosis-associated ovarian carcinomas: how PI3K/AKT/mTOR pathway affects their pathogenesis. Biomolecules 13. (2023) 13(8):1253. doi: 10.3390/biom13081253, PMID: 37627318 PMC10452661

[23] Blanc-Durand F Ngoi N Lim D Ray-Coquard I Tan DS. Clearer Horizons: The latest advances in clear cell ovarian cancer treatment. Cancer Treat Rev. (2025) 138:102977. doi: 10.1016/j.ctrv.2025.102977, PMID: 40517636

[24] Meagher NS Köbel M Karnezis AN Talhouk A Anglesio MS Berchuck A. Cellular origins of mucinous ovarian carcinoma. J Pathol. (2025) 266:9–25. doi: 10.1002/path.6407, PMID: 40028669 PMC11985703

[25] Bell D Berchuck A Birrer M Chien J Cramer DW Dao F. Integrated genomic analyses of ovarian carcinoma. Nature. (2011) 474:609–15. doi: 10.1038/nature10166, PMID: 21720365 PMC3163504

[26] da Costa A Baiocchi G. Genomic profiling of platinum-resistant ovarian cancer: The road into druggable targets. Semin Cancer Biol. (2021) 77:29–41. doi: 10.1016/j.semcancer.2020.10.016, PMID: 33161141

[27] Vergote I González-Martín A Ray-Coquard I Harter P Colombo N Pujol P. European experts consensus: BRCA/homologous recombination deficiency testing in first-line ovarian cancer. Ann Oncol. (2022) 33:276–87. doi: 10.1016/j.annonc.2021.11.013, PMID: 34861371

[28] Li S Silvestri V Leslie G Rebbeck TR Neuhausen SL Hopper JL. Cancer risks associated with BRCA1 and BRCA2 pathogenic variants. J Clin Oncol. (2022) 40:1529–41. doi: 10.1200/JCO.21.02112, PMID: 35077220 PMC9084432

[29] Strickland KC Howitt BE Shukla SA Rodig S Ritterhouse LL Liu JF. Association and prognostic significance of BRCA1/2-mutation status with neoantigen load, number of tumor-infiltrating lymphocytes and expression of PD-1/PD-L1 in high grade serous ovarian cancer. Oncotarget. (2016) 7:13587–98. doi: 10.18632/oncotarget.7277, PMID: 26871470 PMC4924663

[30] Bruand M Barras D Mina M Ghisoni E Morotti M Lanitis E. Cell-autonomous inflammation of BRCA1-deficient ovarian cancers drives both tumor-intrinsic immunoreactivity and immune resistance via STING. Cell Rep. (2021) 36:109412. doi: 10.1016/j.celrep.2021.109412, PMID: 34289354 PMC8371260

[31] Webb JR Milne K Kroeger DR Nelson BH. PD-L1 expression is associated with tumor-infiltrating T cells and favorable prognosis in high-grade serous ovarian cancer. Gynecol Oncol. (2016) 141:293–302. doi: 10.1016/j.ygyno.2016.03.008, PMID: 26972336

[32] Park J Kim JC Lee YJ Kim S Seo MK Kim SW. Unique immune characteristics and differential anti-PD-1-mediated reinvigoration potential of CD8(+) TILs based on BRCA1/2 mutation status in epithelial ovarian cancers. J Immunother Cancer. (2024) 12(7):e009058. doi: 10.1136/jitc-2024-009058, PMID: 38964784 PMC11227838

[33] Zhang AW McPherson A Milne K Kroeger DR Hamilton PT Miranda A. Interfaces of Malignant and immunologic clonal dynamics in ovarian cancer. Cell. (2018) 173:1755–1769.e22. doi: 10.1016/j.cell.2018.03.073, PMID: 29754820

[34] Burdett NL Willis MO Alsop K Hunt AL Pandey A Hamilton PT. Multiomic analysis of homologous recombination-deficient end-stage high-grade serous ovarian cancer. Nat Genet. (2023) 55:437–50. doi: 10.1038/s41588-023-01320-2, PMID: 36849657

[35] Mikulak J Terzoli S Marzano P Cazzetta V Martiniello G Piazza R. Immune evasion mechanisms in early-stage I high-grade serous ovarian carcinoma: insights into regulatory T cell dynamics. Cell Death Dis. (2025) 16:229. doi: 10.1038/s41419-025-07557-5, PMID: 40164596 PMC11958665

[36] Meagher NS Hamilton P Milne K Thornton S Harris B Weir A. Profiling the immune landscape in mucinous ovarian carcinoma. Gynecol Oncol. (2023) 168:23–31. doi: 10.1016/j.ygyno.2022.10.022, PMID: 36368129 PMC10374276

[37] Chen S Li Y Qian L Deng S Liu L Xiao W. A review of the clinical characteristics and novel molecular subtypes of endometrioid ovarian cancer. Front Oncol. (2021) 11:668151. doi: 10.3389/fonc.2021.668151, PMID: 34150634 PMC8210668

[38] Bolton KL Chen D Corona de la Fuente R Fu Z Murali R Köbel M. Molecular subclasses of clear cell ovarian carcinoma and their impact on disease behavior and outcomes. Clin Cancer Res. (2022) 28:4947–56. doi: 10.1158/1078-0432.CCR-21-3817, PMID: 35816189 PMC9777703

[39] Locati M Curtale G Mantovani A. Diversity, mechanisms, and significance of macrophage plasticity. Annu Rev Pathol. (2020) 15:123–47. doi: 10.1146/annurev-pathmechdis-012418-012718, PMID: 31530089 PMC7176483

[40] Toledo B Zhu Chen L Paniagua-Sancho M Marchal JA Perán M Giovannetti E. Deciphering the performance of macrophages in tumour microenvironment: a call for precision immunotherapy. J Hematol Oncol. (2024) 17:44. doi: 10.1186/s13045-024-01559-0, PMID: 38863020 PMC11167803

[41] Sica A Mantovani A. Macrophage plasticity and polarization: *in vivo* veritas. J Clin Invest. (2012) 122:787–95. doi: 10.1172/JCI59643, PMID: 22378047 PMC3287223

[42] Gupta V Yull F Khabele D. Bipolar tumor-associated macrophages in ovarian cancer as targets for therapy. Cancers (Basel) 10. (2018) 10(10):366. doi: 10.3390/cancers10100366, PMID: 30274280 PMC6210537

[43] Yousefzadeh Y Hallaj S Baghi Moornani M Asghary A Azizi G Hojjat-Farsangi M. Tumor associated macrophages in the molecular pathogenesis of ovarian cancer. Int Immunopharmacol. (2020) 84:106471. doi: 10.1016/j.intimp.2020.106471, PMID: 32305830

[44] Krishnan V Schaar B Tallapragada S Dorigo O. Tumor associated macrophages in gynecologic cancers. Gynecol Oncol. (2018) 149:205–13. doi: 10.1016/j.ygyno.2018.01.014, PMID: 29395307

[45] Baer C Squadrito ML Laoui D Thompson D Hansen SK Kiialainen A. Suppression of microRNA activity amplifies IFN-γ-induced macrophage activation and promotes anti-tumour immunity. Nat Cell Biol. (2016) 18:790–802. doi: 10.1038/ncb3371, PMID: 27295554

[46] Boutilier AJ Elsawa SF. Macrophage polarization states in the tumor microenvironment. Int J Mol Sci 22. (2021) 22(13):6995. doi: 10.3390/ijms22136995, PMID: 34209703 PMC8268869

[47] Mantovani A Marchesi F Malesci A Laghi L Allavena P. Tumour-associated macrophages as treatment targets in oncology. Nat Rev Clin Oncol. (2017) 14:399–416. doi: 10.1038/nrclinonc.2016.217, PMID: 28117416 PMC5480600

[48] Mantovani A Sozzani S Locati M Allavena P Sica A. Macrophage polarization: tumor-associated macrophages as a paradigm for polarized M2 mononuclear phagocytes. Trends Immunol. (2002) 23:549–55. doi: 10.1016/S1471-4906(02)02302-5, PMID: 12401408

[49] Lin SC Liao YC Chen PM Yang YY Wang YH Tung SL. Correction: Periostin promotes ovarian cancer metastasis by enhancing M2 macrophages and cancer-associated fibroblasts via integrin-mediated NF-κB and TGF-β2 signaling. J BioMed Sci. (2023) 30:54. doi: 10.1186/s12929-023-00948-w, PMID: 37438746 PMC10337140

[50] Lane D Matte I Rancourt C Piché A. Prognostic significance of IL-6 and IL-8 ascites levels in ovarian cancer patients. BMC Cancer. (2011) 11:210. doi: 10.1186/1471-2407-11-210, PMID: 21619709 PMC3118896

[51] Reinartz S Schumann T Finkernagel F Wortmann A Jansen JM Meissner W. Mixed-polarization phenotype of ascites-associated macrophages in human ovarian carcinoma: correlation of CD163 expression, cytokine levels and early relapse. Int J Cancer. (2014) 134:32–42. doi: 10.1002/ijc.28335, PMID: 23784932 PMC4232932

[52] Hensler M Kasikova L Fiser K Rakova J Skapa P Laco J. M2-like macrophages dictate clinically relevant immunosuppression in metastatic ovarian cancer. J Immunother Cancer. (2020) 8(2):e000979. doi: 10.1136/jitc-2020-000979, PMID: 32819974 PMC7443306

[53] Paolini L Adam C Beauvillain C Preisser L Blanchard S Pignon P. Lactic acidosis together with GM-CSF and M-CSF induces human macrophages toward an inflammatory protumor phenotype. Cancer Immunol Res. (2020) 8:383–95. doi: 10.1158/2326-6066.CIR-18-0749, PMID: 31924656

[54] Goossens P Rodriguez-Vita J Etzerodt A Masse M Rastoin O Gouirand V. Membrane cholesterol efflux drives tumor-associated macrophage reprogramming and tumor progression. Cell Metab. (2019) 29:1376–1389.e4. doi: 10.1016/j.cmet.2019.02.016, PMID: 30930171

[55] Izar B Tirosh I Stover EH Wakiro I Cuoco MS Alter I. A single-cell landscape of high-grade serous ovarian cancer. Nat Med. (2020) 26:1271–9. doi: 10.1038/s41591-020-0926-0, PMID: 32572264 PMC7723336

[56] Luo Y Xia Y Liu D Li X Li H Liu J. Neoadjuvant PARPi or chemotherapy in ovarian cancer informs targeting effector Treg cells for homologous-recombination-deficient tumors. Cell. (2024) 187:4905–4925.e24. doi: 10.1016/j.cell.2024.06.013, PMID: 38971151

[57] Cai L Li Y Tan J Xu L Li Y LAG-3 T. TIM-3, and TIGIT for cancer immunotherapy. J Hematol Oncol. (2023) 16:101. doi: 10.1186/s13045-023-01499-1, PMID: 37670328 PMC10478462

[58] Scott AC Dündar F Zumbo P Chandran SS Klebanoff CA Shakiba M. TOX is a critical regulator of tumour-specific T cell differentiation. Nature. (2019) 571:270–4. doi: 10.1038/s41586-019-1324-y, PMID: 31207604 PMC7698992

[59] Khan O Giles JR McDonald S Manne S Ngiow SF Patel KP. TOX transcriptionally and epigenetically programs CD8(+) T cell exhaustion. Nature. (2019) 571:211–8. doi: 10.1038/s41586-019-1325-x, PMID: 31207603 PMC6713202

[60] Seo H Chen J González-Avalos E Samaniego-Castruita D Das A Wang YH. TOX and TOX2 transcription factors cooperate with NR4A transcription factors to impose CD8(+) T cell exhaustion. Proc Natl Acad Sci U.S.A. (2019) 116:12410–5. doi: 10.1073/pnas.1905675116, PMID: 31152140 PMC6589758

[61] Han HS Jeong S Kim H Kim HD Kim AR Kwon M. TOX-expressing terminally exhausted tumor-infiltrating CD8(+) T cells are reinvigorated by co-blockade of PD-1 and TIGIT in bladder cancer. Cancer Lett. (2021) 499:137–47. doi: 10.1016/j.canlet.2020.11.035, PMID: 33249194

[62] Chen J López-Moyado IF Seo H Lio CJ Hempleman LJ Sekiya T. NR4A transcription factors limit CAR T cell function in solid tumours. Nature. (2019) 567:530–4. doi: 10.1038/s41586-019-0985-x, PMID: 30814732 PMC6546093

[63] Lukhele S Rabbo DA Guo M Shen J Elsaesser HJ Quevedo R. The transcription factor IRF2 drives interferon-mediated CD8(+) T cell exhaustion to restrict anti-tumor immunity. Immunity. (2022) 55:2369–2385.e10. doi: 10.1016/j.immuni.2022.10.020, PMID: 36370712 PMC9809269

[64] Zebley CC Gottschalk S Youngblood B. Rewriting history: epigenetic reprogramming of CD8(+) T cell differentiation to enhance immunotherapy. Trends Immunol. (2020) 41:665–75. doi: 10.1016/j.it.2020.06.008, PMID: 32624330 PMC7395868

[65] Sen DR Kaminski J Barnitz RA Kurachi M Gerdemann U Yates KB. The epigenetic landscape of T cell exhaustion. Science. (2016) 354:1165–9. doi: 10.1126/science.aae0491, PMID: 27789799 PMC5497589

[66] Martínez-Zamudio RI Dewald HK Vasilopoulos T Gittens-Williams L Fitzgerald-Bocarsly P Herbig U. Senescence-associated β-galactosidase reveals the abundance of senescent CD8+ T cells in aging humans. Aging Cell. (2021) 20:e13344. doi: 10.1111/acel.13344, PMID: 33939265 PMC8135084

[67] Montes CL Chapoval AI Nelson J Orhue V Zhang X Schulze DH. Tumor-induced senescent T cells with suppressor function: a potential form of tumor immune evasion. Cancer Res. (2008) 68:870–9. doi: 10.1158/0008-5472.CAN-07-2282, PMID: 18245489

[68] Piskorz WM Cechowska-Pasko M. Senescence of tumor cells in anticancer therapy-beneficial and detrimental effects. Int J Mol Sci 23. (2022) 23(19):11082. doi: 10.3390/ijms231911082, PMID: 36232388 PMC9570404

[69] Mondal T Gaur H Wamba BEN Michalak AG Stout C Watson MR. Characterizing the regulatory Fas (CD95) epitope critical for agonist antibody targeting and CAR-T bystander function in ovarian cancer. Cell Death Differ. (2023) 30:2408–31. doi: 10.1038/s41418-023-01229-7, PMID: 37838774 PMC10657439

[70] Upadhyay R Boiarsky JA Pantsulaia G Svensson-Arvelund J Lin MJ Wroblewska A. A critical role for fas-mediated off-target tumor killing in T-cell immunotherapy. Cancer Discov. (2021) 11:599–613. doi: 10.1158/2159-8290.CD-20-0756, PMID: 33334730 PMC7933082

[71] Liu J Liu J Qin G Li J Fu Z Li J. MDSCs-derived GPR84 induces CD8(+) T-cell senescence via p53 activation to suppress the antitumor response. J Immunother Cancer 11. (2023) 11(11):e007802. doi: 10.1136/jitc-2023-007802, PMID: 38016719 PMC10685939

[72] Li L Ma Y Xu Y. Follicular regulatory T cells infiltrated the ovarian carcinoma and resulted in CD8 T cell dysfunction dependent on IL-10 pathway. Int Immunopharmacol. (2019) 68:81–7. doi: 10.1016/j.intimp.2018.12.051, PMID: 30616170

[73] Sato S Matsushita H Shintani D Kobayashi Y Fujieda N Yabuno A. Association between effector-type regulatory T cells and immune checkpoint expression on CD8(+) T cells in Malignant ascites from epithelial ovarian cancer. BMC Cancer. (2022) 22:437. doi: 10.1186/s12885-022-09534-z, PMID: 35449092 PMC9026673

[74] Fang Z Meng Q Xu J Wang W Zhang B Liu J. Signaling pathways in cancer-associated fibroblasts: recent advances and future perspectives. Cancer Commun (Lond). (2023) 43:3–41. doi: 10.1002/cac2.12392, PMID: 36424360 PMC9859735

[75] Iliopoulos D Hirsch HA Struhl K. An epigenetic switch involving NF-kappaB, Lin28, Let-7 MicroRNA, and IL6 links inflammation to cell transformation. Cell. (2009) 139:693–706. doi: 10.1016/j.cell.2009.10.014, PMID: 19878981 PMC2783826

[76] Bernson E Huhn O Karlsson V Hawkes D Lycke M Cazzetta V. Identification of tissue-resident natural killer and T lymphocytes with anti-tumor properties in ascites of ovarian cancer patients. Cancers (Basel). (2023) 15(13):3362. doi: 10.3390/cancers15133362, PMID: 37444472 PMC10340516

[77] Hornburg M Desbois M Lu S Guan Y Lo AA Kaufman S. Single-cell dissection of cellular components and interactions shaping the tumor immune phenotypes in ovarian cancer. Cancer Cell. (2021) 39:928–944.e6. doi: 10.1016/j.ccell.2021.04.004, PMID: 33961783

[78] Simmons D Makgoba MW Seed B. ICAM, an adhesion ligand of LFA-1, is homologous to the neural cell adhesion molecule NCAM. Nature. (1988) 331:624–7. doi: 10.1038/331624a0, PMID: 3340213

[79] Kvale D Brandtzaeg P. Immune modulation of adhesion molecules ICAM-1 (CD54) and LFA-3 (CD58) in human hepatocytic cell lines. J Hepatol. (1993) 17:347–52. doi: 10.1016/S0168-8278(05)80216-8, PMID: 7686194

[80] Musso A Condon TP West GA de la Motte C Strong SA Levine AD. Regulation of ICAM-1-mediated fibroblast-T cell reciprocal interaction: implications for modulation of gut inflammation. Gastroenterology. (1999) 117:546–56. doi: 10.1016/S0016-5085(99)70447-6, PMID: 10464130

[81] Sans M Panés J Ardite E Elizalde JI Arce Y Elena M. VCAM-1 and ICAM-1 mediate leukocyte-endothelial cell adhesion in rat experimental colitis. Gastroenterology. (1999) 116:874–83. doi: 10.1016/S0016-5085(99)70070-3, PMID: 10092309

[82] Piao W Li L Saxena V Iyyathurai J Lakhan R Zhang Y. PD-L1 signaling selectively regulates T cell lymphatic transendothelial migration. Nat Commun. (2022) 13:2176. doi: 10.1038/s41467-022-29930-0, PMID: 35449134 PMC9023578

[83] Loves R Grunebaum E. FAS signalling pathway is crucial for CAR T cell persistence. Nat Rev Immunol. (2024) 24:380. doi: 10.1038/s41577-024-01038-0, PMID: 38671171

[84] Menegatti S Lopez-Cobo S Sutra Del Galy A Fuentealba J Silva L Perrin L. Ablation of FAS confers allogeneic CD3(-) CAR T cells with resistance to rejection by T cells and natural killer cells. Nat BioMed Eng. (2024) 8:1651–64. doi: 10.1038/s41551-024-01282-8, PMID: 39558141

[85] Morotti M Grimm AJ Hope HC Arnaud M Desbuisson M Rayroux N. PGE(2) inhibits TIL expansion by disrupting IL-2 signalling and mitochondrial function. Nature. (2024) 629:426–34. doi: 10.1038/s41586-024-07352-w, PMID: 38658764 PMC11078736

[86] Pearce OMT Delaine-Smith RM Maniati E Nichols S Wang J Böhm S. Deconstruction of a metastatic tumor microenvironment reveals a common matrix response in human cancers. Cancer Discov. (2018) 8:304–19. doi: 10.1158/2159-8290.CD-17-0284, PMID: 29196464 PMC5837004

[87] Giordano M Decio A Battistini C Baronio M Bianchi F Villa A. L1CAM promotes ovarian cancer stemness and tumor initiation via FGFR1/SRC/STAT3 signaling. J Exp Clin Cancer Res. (2021) 40:319. doi: 10.1186/s13046-021-02117-z, PMID: 34645505 PMC8513260

[88] Song B Chi H Peng G Song Y Cui Z Zhu Y. Characterization of coagulation-related gene signature to predict prognosis and tumor immune microenvironment in skin cutaneous melanoma. Front Oncol. (2022) 12:975255. doi: 10.3389/fonc.2022.975255, PMID: 36059641 PMC9434152

[89] Rahma OE Hodi FS. The intersection between tumor angiogenesis and immune suppression. Clin Cancer Res. (2019) 25:5449–57. doi: 10.1158/1078-0432.CCR-18-1543, PMID: 30944124

[90] Lacher SB Dörr J de Almeida GP Hönninger J Bayerl F Hirschberger A. PGE(2) limits effector expansion of tumour-infiltrating stem-like CD8(+) T cells. Nature. (2024) 629:417–25. doi: 10.1038/s41586-024-07254-x, PMID: 38658748 PMC11078747

[91] Luo S Yang G Ye P Cao N Chi X Yang WH. Macrophages are a double-edged sword: molecular crosstalk between tumor-associated macrophages and cancer stem cells. Biomolecules 12. (2022) 12(6):850. doi: 10.3390/biom12060850, PMID: 35740975 PMC9221070

[92] Yuan H Qiu Y Mei Z Liu J Wang L Zhang K. Cancer stem cells and tumor-associated macrophages: Interactions and therapeutic opportunities. Cancer Lett. (2025) 624:217737. doi: 10.1016/j.canlet.2025.217737, PMID: 40274063

[93] Wang Y Zong X Mitra S Mitra AK Matei D Nephew KP. IL-6 mediates platinum-induced enrichment of ovarian cancer stem cells. JCI Insight 3. (2018) 3(23):e122360. doi: 10.1172/jci.insight.122360, PMID: 30518684 PMC6328027

[94] Fang Y Xiao X Wang J Dasari S Pepin D Nephew KP. Cancer associated fibroblasts serve as an ovarian cancer stem cell niche through noncanonical Wnt5a signaling. NPJ Precis Oncol. (2024) 8:7. doi: 10.1038/s41698-023-00495-5, PMID: 38191909 PMC10774407

[95] Ferri-Borgogno S Zhu Y Sheng J Burks JK Gomez JA Wong KK. Spatial transcriptomics depict ligand-receptor cross-talk heterogeneity at the tumor-stroma interface in long-term ovarian cancer survivors. Cancer Res. (2023) 83:1503–16. doi: 10.1158/0008-5472.CAN-22-1821, PMID: 36787106 PMC10159916

[96] Wang Y Xiang Y Xin VW Wang XW Peng XC Liu XQ. Dendritic cell biology and its role in tumor immunotherapy. J Hematol Oncol. (2020) 13:107. doi: 10.1186/s13045-020-00939-6, PMID: 32746880 PMC7397618

[97] Chen X Chi H Zhao X Pan R Wei Y Han Y. Role of exosomes in immune microenvironment of hepatocellular carcinoma. J Oncol. (2022) 2022:2521025. doi: 10.1155/2022/2521025, PMID: 35126514 PMC8816547

[98] Gardner GJ Chi DS. Recurrent ovarian cancer - sculpting a promising future with surgery. N Engl J Med. (2021) 385:2187–8. doi: 10.1056/NEJMe2116353, PMID: 34874635

[99] Miras I Estévez-García P Muñoz-Galván S. Clinical and molecular features of platinum resistance in ovarian cancer. Crit Rev Oncol Hematol. (2024) 201:104434. doi: 10.1016/j.critrevonc.2024.104434, PMID: 38960218

[100] Ponzone R. BRCA1/2 status and chemotherapy response score to tailor ovarian cancer surgery. Crit Rev Oncol Hematol. (2021) 157:103128. doi: 10.1016/j.critrevonc.2020.103128, PMID: 33137578

[101] Gao Y Li Y Zhang C Han J Liang H Zhang K. Evaluating the benefits of neoadjuvant chemotherapy for advanced epithelial ovarian cancer: a retrospective study. J Ovarian Res. (2019) 12:85. doi: 10.1186/s13048-019-0562-9, PMID: 31519183 PMC6744704

[102] Mirza MR Coleman RL González-Martín A Moore KN Colombo N Ray-Coquard I. The forefront of ovarian cancer therapy: update on PARP inhibitors. Ann Oncol. (2020) 31:1148–59. doi: 10.1016/j.annonc.2020.06.004, PMID: 32569725

[103] Ray-Coquard I Leary A Pignata S Cropet C González-Martín A Marth C. Olaparib plus bevacizumab first-line maintenance in ovarian cancer: final overall survival results from the PAOLA-1/ENGOT-ov25 trial. Ann Oncol. (2023) 34:681–92. doi: 10.1016/j.annonc.2023.05.005, PMID: 37211045

[104] Gomez-Roca CA Italiano A Le Tourneau C Cassier PA Toulmonde M D’Angelo SP. Phase I study of emactuzumab single agent or in combination with paclitaxel in patients with advanced/metastatic solid tumors reveals depletion of immunosuppressive M2-like macrophages. Ann Oncol. (2019) 30:1381–92. doi: 10.1093/annonc/mdz163, PMID: 31114846 PMC8887589

[105] Colombo N Zaccarelli E Baldoni A Frezzini S Scambia G Palluzzi E. Multicenter, randomised, open-label, non-comparative phase 2 trial on the efficacy and safety of the combination of bevacizumab and trabectedin with or without carboplatin in women with partially platinum-sensitive recurrent ovarian cancer. Br J Cancer. (2019) 121:744–50. doi: 10.1038/s41416-019-0584-5, PMID: 31537908 PMC6888836

[106] Monk BJ Brady MF Aghajanian C Lankes HA Rizack T Leach J. A phase 2, randomized, double-blind, placebo- controlled study of chemo-immunotherapy combination using motolimod with pegylated liposomal doxorubicin in recurrent or persistent ovarian cancer: a Gynecologic Oncology Group partners study. Ann Oncol. (2017) 28:996–1004. doi: 10.1093/annonc/mdx049, PMID: 28453702 PMC5406764

[107] Sikic BI Lakhani N Patnaik A Shah SA Chandana SR Rasco D. First-in-human, first-in-class phase I trial of the anti-CD47 antibody hu5F9-G4 in patients with advanced cancers. J Clin Oncol. (2019) 37:946–53. doi: 10.1200/JCO.18.02018, PMID: 30811285 PMC7186585

[108] Cristescu R Aurora-Garg D Albright A Xu L Liu XQ Loboda A. Tumor mutational burden predicts the efficacy of pembrolizumab monotherapy: a pan-tumor retrospective analysis of participants with advanced solid tumors. J Immunother Cancer 10. (2022) 10(1):e003091. doi: 10.1136/jitc-2021-003091, PMID: 35101941 PMC8804694

[109] Lee EK Xiong N Cheng SC Barry WT Penson RT Konstantinopoulos PA. Combined pembrolizumab and pegylated liposomal doxorubicin in platinum resistant ovarian cancer: A phase 2 clinical trial. Gynecol Oncol. (2020) 159:72–8. doi: 10.1016/j.ygyno.2020.07.028, PMID: 32771276

[110] Moore KN Bookman M Sehouli J Miller A Anderson C Scambia G. Atezolizumab, bevacizumab, and chemotherapy for newly diagnosed stage III or IV ovarian cancer: placebo-controlled randomized phase III trial (IMagyn050/GOG 3015/ENGOT-OV39). J Clin Oncol. (2021) 39:1842–55. doi: 10.1200/JCO.21.00306, PMID: 33891472 PMC8189598

[111] Moore KN Pignata S. Trials in progress: IMagyn050/GOG 3015/ENGOT-OV39. A Phase III, multicenter, randomized study of atezolizumab versus placebo administered in combination with paclitaxel, carboplatin, and bevacizumab to patients with newly-diagnosed stage III or stage IV ovarian, fallopian tube, or primary peritoneal cancer. Int J Gynecol Cancer. (2019) 29:430–3. doi: 10.1136/ijgc-2018-000071, PMID: 30630885

[112] Vergote I Heitz F Buderath P Powell M Sehouli J Lee CM. A randomized, double-blind, placebo-controlled phase 1b/2 study of ralimetinib, a p38 MAPK inhibitor, plus gemcitabine and carboplatin versus gemcitabine and carboplatin for women with recurrent platinum-sensitive ovarian cancer. Gynecol Oncol. (2020) 156:23–31. doi: 10.1016/j.ygyno.2019.11.006, PMID: 31791552

[113] Tap WD Gelderblom H Palmerini E Desai J Bauer S Blay JY. Pexidartinib versus placebo for advanced tenosynovial giant cell tumour (ENLIVEN): a randomised phase 3 trial. . Lancet. (2019) 394:478–87. doi: 10.1016/S0140-6736(19)30764-0, PMID: 31229240 PMC6860022

[114] Durham BH Lopez Rodrigo E Picarsic J Abramson D Rotemberg V De Munck S. Activating mutations in CSF1R and additional receptor tyrosine kinases in histiocytic neoplasms. Nat Med. (2019) 25:1839–42. doi: 10.1038/s41591-019-0653-6, PMID: 31768065 PMC6898787

[115] De Henau O Rausch M Winkler D Campesato LF Liu C Cymerman DH. Overcoming resistance to checkpoint blockade therapy by targeting PI3Kγ in myeloid cells. Nature. (2016) 539:443–7. doi: 10.1038/nature20554, PMID: 27828943 PMC5634331

[116] Dudek AZ Yunis C Harrison LI Kumar S Hawkinson R Cooley S. First in human phase I trial of 852A, a novel systemic toll-like receptor 7 agonist, to activate innate immune responses in patients with advanced cancer. Clin Cancer Res. (2007) 13:7119–25. doi: 10.1158/1078-0432.CCR-07-1443, PMID: 18056192

[117] Bourquin C Pommier A Hotz C. Harnessing the immune system to fight cancer with Toll-like receptor and RIG-I-like receptor agonists. Pharmacol Res. (2020) 154:104192. doi: 10.1016/j.phrs.2019.03.001, PMID: 30836160

[118] Song B Wu P Liang Z Wang J Zheng Y Wang Y. A novel necroptosis-related gene signature in skin cutaneous melanoma prognosis and tumor microenvironment. Front Genet. (2022) 13:917007. doi: 10.3389/fgene.2022.917007, PMID: 35899194 PMC9309482

[119] Chi H Huang J Yan Y Jiang C Zhang S Chen H. Unraveling the role of disulfidptosis-related LncRNAs in colon cancer: a prognostic indicator for immunotherapy response, chemotherapy sensitivity, and insights into cell death mechanisms. Front Mol Biosci. (2023) 10:1254232. doi: 10.3389/fmolb.2023.1254232, PMID: 37916187 PMC10617599

[120] Huang X Chi H Gou S Guo X Li L Peng G. An aggrephagy-related lncRNA signature for the prognosis of pancreatic adenocarcinoma. Genes (Basel). (2023) 14(1):124. doi: 10.3390/genes14010124, PMID: 36672865 PMC9859148

[121] Pang Y Hou X Yang C Liu Y Jiang G. Advances on chimeric antigen receptor-modified T-cell therapy for oncotherapy. Mol Cancer. (2018) 17:91. doi: 10.1186/s12943-018-0840-y, PMID: 29769134 PMC5956614

[122] Launonen IM Vähärautio A Färkkilä A. The emerging role of the single-cell and spatial tumor microenvironment in high-grade serous ovarian cancer. Cold Spring Harb Perspect Med. (2023) 13:a041314. doi: 10.1101/cshperspect.a041314, PMID: 37553211 PMC10547388

